# Histological classification and expression of markers of canine mast cell tumors

**DOI:** 10.14202/vetworld.2020.1627-1634

**Published:** 2020-08-18

**Authors:** V. S. Cruz, J. C. A. Borges, L. L. Nepomuceno, P. A. M. Gonçalves, Y. C. L. Prado, C. Bianchi, M. C. S. Fioravanti, E. G. Araújo

**Affiliations:** 1 Multi-User Laboratory for the Evaluation of Molecules Cells and Tissues, Veterinary and Zootechnical School of the Federal University of Goiás, Campus Samambaia, Avenida Esperança, Goiânia, GO 74690-900, Brazil; 2 Department of Veterinary Medicine of the University Center Nossa Senhora do Patrocínio, Pc Antônio Vieira Tavares, 73, Salto - SP, 13320-219, Brazil; 3 Department of Experimental Medicine of the University of Mogi das Cruzes, Av. Dr. Cândido X. de Almeida e Souza, 200 - Centro Cívico, Mogi das Cruzes - SP, 08780-911, Brazil

**Keywords:** Bismarck brown, hematoxylin and eosin, ImageJ, round cell tumor, toluidine blue

## Abstract

**Background and Aim:**

Mast cell tumors (MCTs) are malignant neoplasms that are common in dogs. Their biological behavior is variable and unpredictable. The aim of the present study was to analyze the histological classification and expression of markers of canine MCTs.

**Materials and Methods:**

Thirty samples of canine MCTs were graded according to the histological classification methods of Patnaik and those of Kiupel. The expression of phosphoprotein 53 (p53) and c-kit proteins was quantified by immunohistochemistry using image processing software, ImageJ - a public domain computer program, developed at the National Institutes of Health.

**Results:**

It was possible to determine the grade of 100% of the samples. According to Patnaik’s classification, 20.00% of the samples were Grade 1, 43.30% were Grade 2, and 36.70% were Grade 3. According to Kiupel’s classification, 56.67% of the samples were of high intensity and 43.33% were of low intensity. Grade 1 tumors had the highest expression of p53 and c-kit, and Grade 2 had the lowest expression. The results showed that it is necessary to perform both histological grading methods. The classification into high and low intensity may provide more consistent results than the three-level grading system. However, a smaller number of categories, although it facilitates the classification, may not be sufficient for the prognosis.

**Conclusion:**

Quantitative evaluation of p-53 and c-kit expression is a useful tool to increase the accuracy of the analysis and to aid in choosing the treatment method for canine MCTs. Histological grading should be combined with other diagnostic methods.

## Introduction

Mast cell tumor (MCT) is a malignant neoplasm of cutaneous or visceral origin that is common in dogs. Its behavior is variable and unpredictable, and it often metastasizes, especially to the lymph nodes, skin, spleen, liver, and bone marrow [1-3]. Histological classification of MCTs is used routinely for estimating the prognosis and choosing therapeutic management [4].

The subjectivity of histological grading of canine MCTs has been contested since 1989. One of the grading systems adopted is the method defined by Patnaik, which describes Grade 1 as well-differentiated tumors, Grade 2 as intermediate tumors, and Grade 3 as poorly differentiated tumors with more aggressive biological behavior. In 2011, Kiupel *et al*. [5] proposed a new grading system that divides tumors into two classes, low intensity and high intensity. A tumor is classified as high intensity when all of the following criteria are observed in 10 analyzed fields: Seven mitotic figures, three multinucleated cells, and three atypical nuclei [5-7]. A possible cause of MCT is the c-kit mutation. The c-kit proto-oncogene product is used as a marker for the diagnosis and prognosis of MCT. Measurement of the expression of the c-kit proto-oncogene by immunohistochemistry is a valuable method for the detection of MCT [8-10]. Another likely cause of MCT is mutation of the tumor suppressor gene tumor protein 53 (TP53), which regulates an extensive network protecting the integrity of the genome from damage and encodes phosphoprotein 53 (p53). Inactivation of the p53 pathway in cancer often results from the occurrence of the mutant p53 protein, which is associated with the worst disease-free survival and has been implicated in resistance to anticancer therapies. Expression of TP53, as detected by immunohistochemistry, indicates a poor prognosis [11-13].

The aim of the present study was to analyze the histological classification and expression of tumor markers of canine MCTs.

## Materials and Methods

### Ethical approval

Not applicable, this study did not need approval from the ethics committee because it was made in paraffin blocks from the Pathology Department file, did not use animals or data from the animals’ medical records.

### Study period and location

The study was carried out in the Pathology Department and in the Multiuser Laboratory for the Evaluation of Molecules, Cells and Tissues of the School of Veterinary and Zootechnics of the Federal University of Goiás, from 2010 to 2015.

### Materials studied

This study aimed to complement the grading methods through the use of histological, histochemical, and immunohistochemical techniques, to improve the accuracy of the diagnosis. Therefore, we did not gather epidemiological and clinical information for the evaluation of canine MCTs.

The study was performed with 30 samples of canine MCTs, previously inserted into paraffin, selected from the Laboratory of Animal Pathology of the School of Veterinary and Animal Science of the Universidade Federal de Goiás. The fragments were cut into 5 μm thick sections by an automatic microtome, and the sections adhered to histology slides impregnated with 3% organosilane solution (aminopropyltriethoxysilane; Sigma, St. Louis, MO, USA) in acetone for immunohistochemical analysis.

### Techniques

MCTs were graded according to the Patnaik [7] and Kiupel [5] systems by histological and histochemical methods using hematoxylin and eosin, ­toluidine blue, and Bismarck brown stains. Routine laboratory protocols were used for hematoxylin and eosin and toluidine blue stains [14]. The technique used for Bismarck brown staining was the method described by Leach [15], which started with dewaxing followed by the rehydration phase. The fragments were treated by dripping a solution containing hydrochloric acid and 70% alcohol for 2 min. The fragments were stained with Bismarck Brown, a solution containing Bismarck Brown (Vesuvina; Labimpex, Diadema, Brazil), ferric chloride (P.A.ACS; Labimpex, Diadema, Brazil), and 70% alcohol, for 60 min. The sections were washed with 70% alcohol, stained with Harris hematoxylin for 3 min, washed with running water for 8 min, and dehydrated. The slides were mounted with a toluene-based medium (Entellan; Merck, Germany) and histological coverslips. The slides were examined by three pathologists under an optical microscope.

The immunohistochemistry procedure started with dewaxing in an oven at 60°C, following the hydration phase. Endogenous peroxidase activity was then blocked for 15 min, and the slides were incubated with dilute hydrogen peroxide in phosphate-buffered saline (PBS) solution (pH 7.2). Antigen retrieval occurred in citrate solution (pH 6.0) in a bain-marie (solution preheated to 96°C) for 15 min. Non-specific binding was blocked with 3% bovine serum albumin (BSA) for 1 h in a humid chamber at room temperature.

The primary antibodies p53 (monoclonal/mouse, SC71785; Santa Cruz Biotechnology, Dallas, TX, USA) and c-kit (polyclonal/rabbit, SC168; Santa Cruz Biotechnology), diluted in 1.5% BSA (1:500 and 1:2000, respectively), were instilled, and the material was incubated overnight in a humid chamber under refrigeration at 4°C. Negative controls were performed by omitting the primary antibody in a sample section of each grade.

The sections were washed with PBS and then incubated at room temperature using the LSAB kit (DAKO, K0690-USA), where the corresponding secondary antibody remained for 30 min and the streptavidin–biotin–peroxidase complex remained for a further 30 min. They were then washed with PBS. The reaction was developed by adding the chromogen diaminobenzidine peroxidase (Liquid DAB Substrate Chromogen System; DAKO Ref: K3468-USA) for 10 min in the sections treated with anti-p53 antibody and 3 min in the sections treated with anti-c-kit antibody.

The slides were mounted with a toluene-based medium (Entellan) and histological coverslips. The analysis was performed by three pathologists under a light microscope. Three areas of concentration of neoplastic cells were selected, according to the availability of material regarding the histological grade, such as regions of interest for evaluation using the ImageJ image processor (NIH, Bethesda, MD, USA). The software provided the optical density values for the selected areas, expressed in pixels/inch.

### Statistical analysis

The non-parametric Chi-square test was used to compare the number of cases diagnosed using the combination of hematoxylin and eosin and toluidine blue stains with the number of diagnoses after the addition of Bismarck brown stain.

The results of the ImageJ image processor were transferred to a spreadsheet. In each group, the median and mean values for each antibody were calculated to represent the central tendency, the 95% confidence interval by logistic regression, and the confidence interval limit. Slides with optical density values below the lower limit, established by the difference between the mean and the confidence interval, received a score of 1; slides with values between the lower limit and the upper limit received a score of 2; and slides with values above the upper limit, established by the sum of the mean and the confidence interval, received a score of 3.

## Results

**Figure-1:**
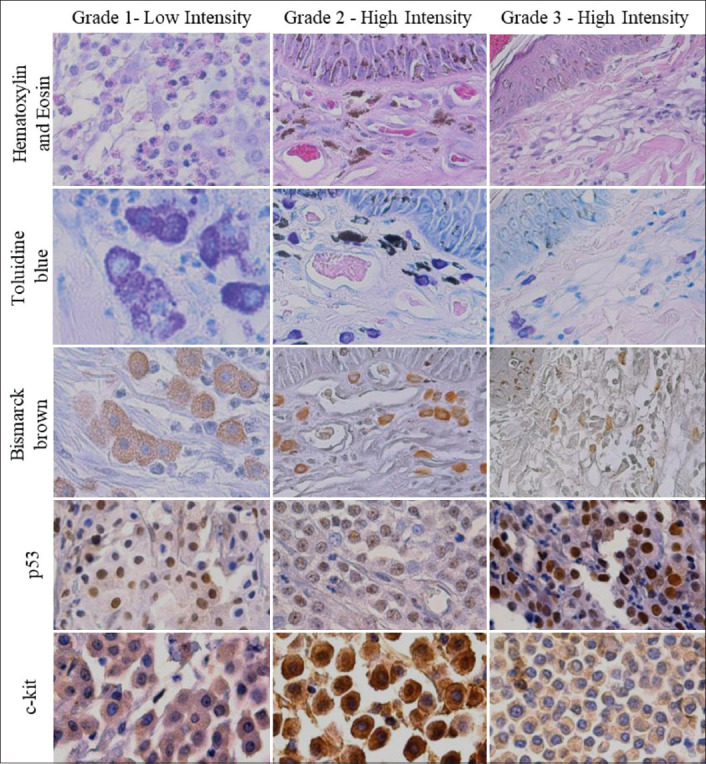
Photomicrographs of canine mast cell tumors representing the histological grading using the Patnaik and Kiupel methods. Hematoxylin and eosin, toluidine blue and Bismarck brown stains. Antibody markers: Anti-p53 in the nucleus and anti-c-kit in the cytoplasm of neoplastic mast cells. DAB, counterstained with hematoxylin, original 400×.

Using hematoxylin and eosin associated with toluidine blue stain, we were able to grade 73.33% (22/30) of the MCTs using the Patnaik system. After using Bismarck brown stain, we were able to grade 100% (30/30) of the MCTs. The incidence and frequency of the main microscopic changes found in each grade are described in Table-1. Thus, with the use of Bismarck brown stain, 20.00% of the samples were classified as Grade 1, 43.30% as Grade 2, and 36.70% as Grade 3. The representative board with the three colors in each grade is shown in Figure-1.

**Table-1:** Incidence and frequency of main microscopic changes found in each grade of canine mast cell tumors through histochemical evaluation, using hematoxylin and eosin, toluidine blue the Bismarck brown stains.

Grade	Location	Cell size	Cell shape	Cytoplasm	Nucleus	Nucleolus	Metachromasia
1 n=6	Superficial Dermis 6 (100%)	Uniform 6 (100%)	Round to oval 6 (100%)	Abundant 6 (100%)	Round to oval 6 (100%)	1 or +/ cell 3 (50%)	Marked 5 (83.33%)
						1/cell 3 (50%)	Moderate 1 (16.67%)
2 n=13	Superficial Dermis 10 (76.90%) Deep Dermis 3 (23.10%)	Anisocytosis and multinucleated cells 9 (69.20%) Uniform 4 (30.80%)	Pleomorphic 10 (76.90%) Round to oval 3 (23.10%)	Moderate 10 (76.90%) Scarce 2 (15.40%) Abundant 1 (7.70%)	Round to oval 9 (69.20%) Anisokaryosis 4 (30.80%)	1 or +/ cell 9 (69.20%) 1/cell 4 (30.80%)	Marked 7 (53.80%) Moderate 6 (46.20%)
3 n=11	Deep Dermis 8 (72.70%) Superficial Dermis 3 (27.30%)	Anisocytosis and multinucleated cells 8 (72.70%) Uniform 3 (27.30%)	Pleomorphic 7 (63.60%) Round to oval 4 (36.40%)	Scarce 7 (63.60%) Moderate 4 (36.40%)	Round to oval 8 (72.70%) Anisokaryosis 3 (27.30%)	1 or +/ cell 11 (100%)	Orthochromasia 8 (72.70%) Moderate 3 (27.30%)

Subsequently, the samples were reexamined and graded according to the classification system proposed by Kiupel. Among the 30 samples, all 6 samples (100%) classified as Grade 1 by the Patnaik system were reclassified as low-intensity MCTs. Of the 13 samples classified as Grade 2, 9 (69.23%) were reclassified as low-intensity MCTs. Four Grade 2, samples (30.77%) were reclassified as high intensity for having at least seven mitotic figures, three multinucleated cells and three atypical nuclei in the 10 fields analyzed.

Of the 11 samples classified as Grade 3, 9 (81.82%) showed characteristics of high intensity, with at least seven mitotic figures, three multinucleated cells, three atypical nuclei in the 10 fields analyzed, and in addition, karyomegaly was also found. However, 2 samples (18.18%) were reclassified as low intensity, because despite the infiltrative aspect, which would be expected as a characteristic of greater malignancy, few multinucleated cells and no mitotic figures were observed.

According to immunohistochemistry, sections of samples treated with anti-p53 antibody showed marking evidence in the nucleus of neoplastic mast cells and, in rare cases, cytoplasmic marking. Sections from samples treated with anti-c-kit antibody showed cytoplasmic marking of neoplastic mast cells (Figure-1).

Table-2 shows the mean, median, standard deviation, confidence interval, lower limit, and upper limit values, calculated from the output of the ImageJ image processor.

**Table-2:** Analysis of optical density values from the image processor ImageJ, expressed in pixels/inch, in the selected areas of each slide with fragments of canine MCT, marked with antibodies p53 and c-kit by the immunohistochemistry technique.

Analysis	P53	C-kit
Mean	108,777.73	185,222.56
Median	66,128.75	105,181.75
Minimum value	1344.50	1556.00
Maximum value	361,825.80	651,463.30
Standard deviation	108,762.48	187,566.59
Confidence interval	38,919.44	67,118.61
Lower limit	69,858.28	118,103.94
Upper limit	147,697.17	252,341.17

The MCT samples were classified into scores, after evaluating the mean values of the three areas analyzed in each slide marked with the antibodies to p53 and c-kit using the ImageJ image processor. Table-3 shows the results obtained in this study.

**Table-3:** Results of the analysis of 30 samples of canine MCT, obtained by the histological grading proposed by Patnaik as Grade 1 (G1), Grade 2 (G2), and Grade 3 (G3); histological grading proposed by Kiupel as low intensity (LI) and high intensity (HI); expression of p53 and c-kit in optical density from ImageJ expressed in pixels/inch; score calculated after statistical evaluation.

Sample	Patnaik	Kiupel	P53		C-kit	
	
			Optical density	Score	Optical density	Score
1	G1	BI	194,599.00	3	107,132.50	1
2	G1	BI	246,391.50	3	523,545.50	3
3	G1	BI	281,239.00	3	447,620.00	3
4	G1	BI	360,827.50	3	103,231.00	1
5	G1	BI	187,505.00	3	424,692.50	3
6	G1	BI	220,203.00	3	546,866.50	3
7	G2	BI	1344.50	1	19,153.50	1
8	G2	BI	2508.00	1	12,267.00	1
9	G2	AI	14,813.00	1	476,762.50	3
10	G2	AI	18,796.50	1	323,161.50	3
11	G2	BI	56,299.50	1	1556.00	1
12	G2	BI	68,255.50	1	100,491.00	1
13	G2	BI	4982.00	1	60,581.00	1
14	G2	BI	5905.50	1	82,870.67	1
15	G2	BI	22,926.00	1	6691.66	1
16	G2	BI	24,455.00	1	2961.33	1
17	G2	BI	12,964.00	1	32,624.00	1
18	G2	AI	248,498.00	3	52,086.00	1
19	G2	AI	119,783.70	2	2723.33	1
20	G3	AI	48,381.60	1	262,362.30	3
21	G3	AI	23,876.20	1	140,430.80	2
22	G3	AI	107,073.80	2	75,093.25	1
23	G3	AI	124,737.60	2	651,463.30	3
24	G3	AI	111,257.40	2	239,458.00	2
25	G3	AI	74,872.80	1	169,299.30	2
26	G3	BI	53,813.00	1	102,600.30	1
27	G3	BI	64,002.00	1	107,226.30	1
28	G3	AI	15,213.83	1	209,967.80	2
29	G3	AI	361,825.80	3	68,478.00	1
30	G3	AI	185,981.70	3	203,280.00	2

## Discussion

Of the total number of MCTs evaluated using the Patnaik system, 26.67% could not be graded with hematoxylin and eosin and toluidine blue stains. According to Bostock *et al*. [6], well-differentiated MCTs are easier to diagnose in routine histological preparations.

Grading of 100% of the samples was only possible with the use of the Bismarck brown stain. The slides stained by this technique showed marked metachromasia in Grade 1 MCTs, moderate metachromasia in Grade 2 MCTs, and orthochromasia in most Grade 3 MCTs. Metachromasia was found in 73.33% (22/30) of samples stained with hematoxylin and eosin and toluidine blue. The interaction of basic stains with cytoplasmic granules is related to the degree of cell differentiation, because there is a decrease in the sulfation of granules in neoplastic mast cells, making them less metachromatic [16]. According to Hosseini *et al*. [17], the ­presence of cytoplasmic granules assisted in the identification of mast cell subtypes in dogs and humans. This finding is consistent with Navarro [18] who suggested that, due to the presence of acid mucopolysaccharides in the cytoplasm of mast cells, preparations stained by hematoxylin and eosin do not provide a good visualization of these granules, and therefore, the use of other stains is necessary for grading.

Use of the Bismarck brown stain made it easier to diagnose poorly differentiated MCTs, which could be confused with other round cell tumors. Lavalle *et al*. [19] stated that the presence of granules differentiates MCTs from other round cell tumors and that the more undifferentiated cells have fewer granules, which requires the use of special stains in these cases. Hosseini *et al*. [17] reported that the histological grading system was useful to distinguish neoplasms from other malignancies with similar morphology. The reason that the Bismarck brown stain performs well is probably that it does not use water in steps subsequent to fixation since mast cells are poorly visualized in aqueous solutions [15].

For comparative purposes, samples previously classified by the Patnaik method were reexamined and graded according to the grading system proposed by Kiupel. According to the Kiupel system, 56.17% (17/30) of samples were of low intensity and 43.33% (13/30) were of high intensity. Śmiech *et al*. [20] classified 75.8% of MCTs as low intensity and 24.2% as high intensity. These results were similar to those of Hergt *et al*. [21] who classified 74.46% of MCTs as low intensity and 25.54% as high intensity.

Concordance was found between Grade 1 and low intensity because the neoplastic cells were well differentiated, resembling normal mast cells, being monomorphic, and arranged in cords and/or thin layers. In addition, the nuclei were round or oval and small, with almost invisible nucleoli. These characteristics are more apparent to pathologists, favoring the assignment to Grade 1 [22]. In agreement with our results, all Grade 1 MCTs were classified as low intensity by Stefanello *et al*. [23].

As expected, Grade 2 tumors were divided into low- and high-intensity malignancies, showing their biological heterogeneity. This result corroborates the findings of Bostock *et al*. [6], Seguin *et al*. [24], and Balsimelli [25], due to the characteristics of this grade in the system proposed by Patnaik *et al*. [7]. In the study of Stefanello *et al*. [23], 83.5% of Grade 2 MCTs were classified as low intensity. There is a tendency among pathologists to choose Grade 2 when dealing with the limits between Grades 1 and 3 [5]. There is a discrepancy among pathologists regarding the Patnaik classification system, especially with respect to tumors classified as Grade 2 [26]. As a consequence, therapeutic and prognostic management based on histological classification, especially for MCTs of intermediate grade, is highly questionable [27,28].

Among the Grade 3 samples, contrary to expectations, two were reclassified as low intensity, despite the expected infiltrative aspect and observed in these samples.. This result is contrary to the results of Balsimelli [25] and Schlieben [29] and Stefanello *et al*. [23], in which all Grade 3 tumors were reclassified as high intensity.

The results of histopathological classification of MCTs into two levels were more consistent than the results with the three-level classification system because the two-level system provided a better distinction between high- and low-intensity malignant cellular transformation. Moreover, in this study, we demonstrated the subjectivity of classification of tumors classified as intermediate grade. According to Ramos-Vara *et al*. [30], the new histopathological grading system proposed by Kiupel is more consistent in these cases because the grading criteria are quantitative, leading to a decrease in subjectivity.

Kry and Boston [26] found concordance between 75% of pathologists for Grade 3 and 63% for Grade 1 using the Patnaik system. In contrast, there was concordance between 98% of pathologists using the Kiupel system. Several classification systems have been proposed to classify canine MCTs, and the Patnaik system is still the most widely used. However, because the Kiupel classification is based on the presence of mitotic figures, multinucleated cells, karyomegaly, and atypical nuclei, it has a higher probability of prognostic accuracy [17]. It is important to emphasize that perfect determination of the histological grade of the tumor helps in surgical planning and in the prediction of local recurrence and metastatic potential [31].

The mean expressions of p53 and c-kit in samples graded by the Patnaik system were highest for Grade 1 and lowest for Grade 2. Although p53 is a protein often studied in several neoplasms, there are few reports of its expression in canine MCTs of different histological grades. Other authors did not find a relationship between p53 immunoreactivity and the biological behavior of this neoplasm, and the protein was considered a poor prognostic indicator for the analyzed cases [32,33].

In the present study, however, the score results of the quantitative analysis of p53 expression were similar to those for c-kit in 53.33% (16/30) of samples. The cell marking and location of c-kit have been well ­characterized in canine MCTs, and the intensity of expression is associated with a worse prognosis. It is important to consider that c-kit mutations and abnormal expression of c-kit are related to increase *in vitro* cell proliferation of MCTs [27]. c-kit is a reliable immunohistochemical marker, and a positive relationship is expected between the presence of c-kit mutations and a higher grade of canine MCT [8,9]. The findings of the present study show that the expression of p53 was similar to that of c-kit, indicating that p53 expression can also be used to estimate the prognosis of canine MCTs.

The results of the analysis of antibody scoring for Grade 1 differ from a previous characterization [27], in which MCTs with this histological grade were benign tumors with a good prognosis for which surgical removal would be the only treatment. The high values of p53 and c-kit expression found in Grade 1 raise awareness regarding the extent of disease in the group considered to have the best prognosis. This can be explained in two ways. First, it is possible that this result is due to the small sample size or the fact that values above the confidence limit have occurred in animals with multiple lesions, even though the lesions were well differentiated. Second, it is likely that neoplastic cells in dogs with multiple Grade 1 nodules are different from those in solitary tumors [34]. In addition, MCTs appear to have predilections of malignancy according to anatomical location, dog breed, and age [35,36].

Another key factor is that p53 labeling in benign tumors may indicate more aggressive behavior and mutations of the p53 gene may indicate a progression of neoplasia [37]. For Amagai *et al*. [38], the analysis of c-kit labeling in a single tumor region is probably insufficient. The presence of the c-kit mutation in primary MCTs may indicate a high probability of metastases, suggesting that c-kit labeling indicates tumor progression [8].

With regard to the high scores of the analyzed samples of Grade 1, the previous studies found 33.3% rate of recurrence of neoplasia in tumors of this grade [39]. The expression of tumor suppressor genes, such as p53, may indicate the initial stages of a microscopically imperceptible second tumor at the primary site, although the initial growth may have been ­completely removed [40]. Moreover, intense c-kit cytoplasmic labeling is associated with an increased recurrence rate and decreased survival time [28]. Pizzoni *et al*. [41] did not observe any relation between histological degree and survival time and the interval free of neoplasia progression. Fonseca-Alves *et al*. [42] observed a shorter survival time in dogs with double positivity for c-kit and ki67.

Even without observing the animals after tumor excision, the results obtained in the Grade 1 group suggest that canine MCT should not be considered benign in any histological grade, due to its variable biological behavior and prognosis. The biological behavior of canine MCTs [43] ranges from solitary benign nodules to systemic metastatic tumors, which hinders the accuracy of prognosis and choice of treatment [17]. In addition, our study did not evaluate the presence of metastases, which would be relevant, as dogs with distant metastases had significantly shorter survival than those with regional lymph node metastases [44], which are also a negative prognostic indicator, regardless of histological grade [45,46].

After analyzing the score for Grade 2, we reiterate that the results can be attributed to the fact that the parameters of histological grading by Patnaik are subjective, especially for this grade. Veterinary pathologists may interpret the Patnaik system differently, assigning different grades to the same sample, and for that reason, less subjective and more quantitative methods have better clinical value [34].

In our study, 69.23% (9/13) of Grade 2 samples had the lowest scores, whereas 30.77% (4/13) had scores that varied among antibodies and were classified as high intensity according to the Kiupel system. Zemke *et al*. [47] showed the relationship between higher histopathological grade and labeling intensity in poorly differentiated neoplasms, showing that they should present more significant genetic alterations for increased production and consequently the expression of the proto-oncogene c-kit. This finding corroborates our findings for high-intensity MCTs. High-intensity MCTs have a high rate of recurrence [48]. This result is also comparable with that of Thompson [49] who found an association between the c-kit mutation and higher grade in canine cutaneous MCTs. c-kit mutations are significantly associated with a higher grade of neoplastic malignancy and also higher rates of recurrence and death.

Another hypothesis is that the score values in the Grade 2 group are compatible with the absence of differences in histological parameters between Grades 1 and 2. These grades may not belong to distinct groups, but rather represent the same category [5]. The Patnaik system has been used for several decades as a gold standard for the classification of MCTs, based mainly on qualitative characteristics. However, there has been high discrepancy among observers, mainly due to the subjective criteria used to establish the classification. Relapses, the probability of metastases, and animal survival time are extremely important variables, which, in association with the degree of cell differentiation, make an effective diagnosis necessary. Differences in the histopathological classification of MCTs make the prognostic value and also the choice of treatment highly questionable [50].

In the present study, Grade 3 samples showed the largest numerical variation for sampling, probably these samples represent a broader profile of MCTs. Quantitative evaluation of p53 and c-kit expression showed values compatible with the unfavorable prognosis expected for the group in 36.36% (4/11) of the samples. However, we found a lower score in 18.18% (2/11) of the samples, especially in the samples classified as low intensity by the Kiupel system. Neoplasms of this grade are described as poorly differentiated, potentially aggressive, and having an adverse prognosis [13]. Other authors have stated that dogs with Grade 3 neoplasms are more likely to develop local recurrence [26].

## Conclusion

The results showed that it is necessary to use both of the histological grading methods. The classification of MCTs into low and high intensity may ­provide more consistent results than the three-level grading system. However, a smaller number of categories, although it facilitates the classification, may not be sufficient for the prognosis. Quantitative evaluation of p-53 and c-kit expression is a useful tool to increase the accuracy of the analysis and to aid in choosing the treatment of canine MCTs. Histological grading should be combined with other diagnostic methods.

## Authors’ Contributions

VSC and JCAB were in charge of conception and design of the study, acquisition, analysis, and interpretation of data, drafted, and critically revised the manuscript. LLN contributed to conception and analysis of data. PAMG participated in design of the study and interpretation of data. YCLP contributed to design of the study and interpretation of data. CB, MCSF, and EGA supported to analysis and critically revised the manuscript. All authors read and approved the final manuscript.

## Competing Interests

The authors declare that they have no competing interests.

## Publisher’s Note

Veterinary World remains neutral with regard to jurisdictional claims in published institutional affiliation.
